# Transparent brain tumor detection using DenseNet169 and LIME

**DOI:** 10.1038/s41598-025-13233-7

**Published:** 2025-08-01

**Authors:** Lincy Annet Abraham, Gopinath Palanisamy, Goutham Veerapu

**Affiliations:** School of Electronics Engineering, Vellore Institute of Technology, Vellore, 632014 Tamilnadu India

**Keywords:** Brain tumor detection, Deep learning, DenseNet169, Local Interpretable Model-agnostic Explanations (LIME), Medical imaging, Transfer learning, Biomedical engineering, Cancer imaging, Image processing, Machine learning

## Abstract

A crucial area of research in the field of medical imaging is that of brain tumor classification, which greatly aids diagnosis and facilitates treatment planning. This paper proposes DenseNet169-LIME-TumorNet, a model based on deep learning and an integrated combination of DenseNet169 with LIME to boost the performance of brain tumor classification and its interpretability. The model was trained and evaluated on the publicly available Brain Tumor MRI Dataset containing 2,870 images spanning three tumor types. Dense169-LIME-TumorNet achieves a classification accuracy of 98.78%, outperforming widely used architectures including Inception V3, ResNet50, MobileNet V2, EfficientNet variants, and other DenseNet configurations. The integration of LIME provides visual explanations that enhance transparency and reliability in clinical decision-making. Furthermore, the model demonstrates minimal computational overhead, enabling faster inference and deployment in resource-constrained clinical environments, thereby highlighting its practical utility for real-time diagnostic support. Work in the future should run towards creating generalization through the adoption of a multi-modal learning approach, hybrid deep learning development, and real-time application development for AI-assisted diagnosis.

## Introduction

Brain tumors represent one of the most serious medical conditions, requiring prompt and accurate diagnosis for effective treatment planning. The early diagnosis of the tumor is needed to minimize bad patient outcomes; delay in doing this can cause a serious complication and reduce survival rates. Diagnostic imaging by radiologists using MRI, CT, and other imaging modalities for detecting lesions in brain scans has its share of disadvantages in terms of diagnosis. This time-consuming manual evaluation is subject to human interference, variation, error, or bias because of fluctuating degrees of the radiologist’s expertise. Progressive developments witnessed in AI or deep learning have led to the origin of automated diagnostic technologies, which enhance the accuracy of diagnosing diseases, decrease workload, and also allow for improved decision-making in medical imaging^1^.

The application and performance of deep learning models, in particular CNNs, with respect to image classification have shown great promise when it comes to applications like brain tumor detection. Different Convolutional Neural Network architectures, like VGG, ResNet, and DenseNet, are widely researched because of their ability of learning hierarchical features from complicated datasets^2^. DenseNet169, in specific, has gained a lot of attention, this is attributed to the dense connectivity in layers, establishing direct connections from every layer with the consecutive one. This mechanism, therefore, supports feature propagation, reduces redundancy in the parameters, and thus enhances performance in general^3^. Transfer learning with a fine-tuning strategy with a pre-trained model has really been a game-changer in getting improved results in cancer classification. Transfer learning indeed reduces the time for training and other efficiencies with more accuracy into images when the available labeled medical data is considerably less. Manual interpretation of brain MRI scans requires significant expertise and is inherently time-consuming. The process is further complicated by inter-observer variability, where different radiologists may provide differing assessments for the same image due to subjective interpretation. This variability can lead to inconsistent diagnoses and delayed treatment. Additionally, limited access to trained radiologists in many regions, particularly in low-resource settings, exacerbates diagnostic challenges, underscoring the need for reliable automated tools.

High classification accuracy notwithstanding, deep learning models are often criticized for a lack of interpretability^4^. Explainability supports acceptance of an AI’s decision by medical practitioners and, thus, clinical validation. To aid with this challenge, XAI techniques have been developed for interpreting and providing insight into model predictions. One such approach is the scope of Local Interpretable Model-agnostic Explanations (LIME), which gives prominence to the most influential portions of an input image that contribute to a given classification conclusion^5^. By integrating LIME, the work would attempt to link bridge a gap between high-performance deep learning models and clinical adoption in providing interpretable and reliable results.Deep learning, especially Convolutional Neural Networks (CNNs), has revolutionized medical image analysis by enabling automatic feature extraction from raw images, reducing reliance on handcrafted features. CNNs have proven effective in recognizing complex spatial patterns and anatomical structures in MRI. Moreover, the emergence of transformer-based architectures offers improved modeling of global dependencies and attention mechanisms, enhancing classification accuracy in high-resolution medical imaging. However, their computational cost and lack of interpretability often hinder clinical deployment.

This research proposes an easy-to-use and interpretable brain tumor detection system using DenseNet169-based transfer learning and LIME. The model is trained on MRI scan datasets to classify brain tumors and provide visual explanations for its predictions^3^. By implementing deep learning with explainability, radiologists will be equipped to make more confident and accurate decisions in this domain.

### Limitations and contributions

Even though deep learning has made great strides toward accuracy in tumor classification, some challenges still remain: annotated dataset size, computational complexity, and model predictions subjected to bias. In addition, such systems have real-time efficiency and robustness issues across medical imaging domains. To address the current study limitations: through DenseNet169 transfer learning, they present improvements in accuracy and reductions in training time along with data dependency. They also apply pre-trained LIME to make the proposed system more transparent and interpretable for its clinical validation. The overall vision was to design a reasonably good, efficient, and explainable AI-based brain tumor detection system to aid an accurate dimensional diagnostic decision-making in radiologists^6^.

This research presents an advanced, deep learning-based framework for the detection of tumors in the brain, based on DenseNet169 and LIME (Local Interpretable Model-agnostic Explanations), which allows for reasoning behind its predictions. The main contributions of the present study are:

- **Enhanced brain tumor classification using transfer learning** - Here, the pre-trained model DenseNet169 boosts the classification accuracy of brain tumors while considerably limiting the need for labeled data. Moreover, this approach of transfer learning guarantees effective feature extraction and enhances convergence speed during training.
- **Better Interpretability with LIME** - The embedding of Local Interpretable Model-agnostic Explanations (LIME) will produce visual explanations of the model predictions, thus adding to the transparency and providing a way for the radiologists to understand and validate the decision-making behind it.
- **Clinical Applicability and Robustness** - The proposed system has been built to be robust and interpretable for real-life medical application use. By breaking down challenges of model transparency and performance convergence when applied to MRI scan, the research proposes to act as a useful aide to the radiologists’ diagnostic decision-making process.

Despite many advances in this study, a few limitations remain to be narrated. First, the driving challenge remains high-quality annotated datasets, as deep learning models work better with extensive and diverse datasets. The other limiting factor is computational complexity; the DenseNet169-based model is resource-hogging, making deployment difficult in resource-limited medical centers. Although trained on MRIs , the generalized ability could still be compromised by variations in protocols among hospitals and equipment. Class imbalance within the training dataset may offer bias to model predictions, while a potentially heterogeneous patient demographic would vary the performance on that data. Late real-time implementation would equally become difficult since LIME would need to be tuned for real-world applicability, allowing for interpretability while ensuring that rapid forecasts can still suffer from performance limitations. Future attempts at eliminating these limitations will be highly relevant to constructing a better, clinically reliable brain tumor detection system.

The outline of the paper chapter-wise is as follows. Chapter II is a review of the related literature, while the purpose of Chapter III is to give a brief view of the theoretical framework, key concepts, and methodologies. Chapter IV is going to evaluate the experimental result. Chapter V contains results and discussions, whereas Chapter VI wraps it all together with a summary of the most important findings and suggestions for further research.

## Literature review

The case for integration of a deep learning model in brain tumors detection is matched with interpretability models to try to bolster the doctrine of clinical diagnostics through decision-making support mechanisms. A number of research studies employed DenseNet169 and LIME within this context.

Khan et al.^7^ integrates DenseNet169 with advanced machine learning classifiers for enhanced accuracy in brain tumor detection. DenseNet169 for feature extraction integrates several patterns within medial images. Mutually, classified more devices of machine learning further boosted classifications by refining the features captured. This hybrid approach is more geared to ensuring maximum diagnostic accuracy when compared to other deep learning versions. It cites great improvements in the classification of brain tumors due with deep learning and machine learning combined techniques.

Summarize one of the roles of explainable AI in medical imaging with respect to transparency. They applied LIME explanations to elucidate the behavior of a deep-learning model and generate visual explanations for brain tumor identification from MRI images^8^. This will assist the clinician in understanding the AI predictions made, developing trust in automated diagnostics, and improving the interpretability of the models for application in real-world medicine. Their findings do underscore the significance of explainable AI in clinical decision-making.

Bhatia et al.^9^ summarize one of the roles of explainable AI in medical imaging with respect to transparency. They applied LIME explanations to elucidate the behavior of a deep-learning model and generate visual explanations for brain tumor identification from MRI images. This will assist the clinician in understanding the AI predictions made, developing trust in automated diagnostics, and improving the interpretability of the models for application in real-world medicine. Their findings do underscore the significance of explainable AI in clinical decision-making.

Islam et al.^4^ did a comparison of several transfer learning architectures for the diagnosis of brain tumors. They found DenseNet169 to give significantly better performance. Among all the other models applied, DenseNet169 provided decent results for the detection of brain tumors. It improved accuracy by leveraging pre-trained criteria for diagnostics. Their findings also bring home the implications of transfer learning in the improvement of medical image classification. This study concludes that DenseNet169 may be a possible reliable and efficient tool for the detection of brain tumors.

Mukherkjee et al.^10^ combined DenseNet169 and explainable AI techniques, like GradCAM and LIME, to improve the interpretability of brain tumor detection. This approach attempts to make improvements to the interpretation of the model, providing visual explanations for brain tumor detection. Its aim was to raise diagnostic accuracy while ensuring confidence in the current treatment paradigm for AI-aided deliberations. The authors endorsed the argument for explainability as a means for achieving early diagnosis and better patient outcome. The study underscored the significance of the role of AI in enabling reliable and transparent medical imaging solutions.

Kibriya et al.^11^ built a hybrid model combining Vision Transformers (ViT) and Convolutional Neural Networks (CNN) to detect brain tumors. This approach capitalized on ViT’s power to grasp global contextual features alongside CNN’s potential to expound local features. Their work proved that their fusion of both architectures led to improved performance of MRI Classification. Through competent reasoning over both spatial and contextual information, the model promoted a better accuracy in diagnostic techniques. The findings demonstrate the growing potential of hybrid deep learning models in medical image application.

Nanda et al.^12^ For the brain tumor classification into three categories, the possible classification using transfer learning fine-tuning based on EfficientNets has been proposed by Nanda et al. EfficientNets have undoubtedly been revealed to extract important features therefore promoting great diagnostic identifiable possibilities. Their research findings underlined that transfer learning is indeed an imperative error in the enhancement of brain tumor detection. The study also stressed the contribution of EfficientNets towards reliable and efficient medical diagnostics.

Mehrotra et al.^13^ Computer-aided diagnosis systems for multi-class brain tumor classification using six pre-trained deep learning models were developed by Mehrotra et al. A plethora of sophisticated pre-processing and data augmentation techniques were performed to acquire the model-whole training performance. This improvement promoted good computational speed and ensured further refinement of diagnostic accuracy. For certain types of brain tumors, the proposed framework showed very good accuracy, thereby proving the robustness of deep learning techniques in the domain of medical imaging. Their research further emphasized on effective pre-processing being able to enhance AI diagnostic capabilities.

Rahman et al.^14^ showed application of AI in neuro-oncology by establishing explainable AI techniques to diagnose brain tumors through MRI scanning. The model is interpretably increased such that clinicians will realize the approach made by AI on predictions. It emphasized the need for transparency in medical diagnosis to build trust in AI-based decision-making. The research, while improving the accuracy of brain tumor classification, added a significant leap in interpretability . Their findings underscore the key position of explainable AI that further enhances interpretation reliability.

Recent advances in Vision Transformers (ViTs) have shown strong performance in medical imaging tasks due to their ability to capture global contextual features. For instance,^15^used a hybrid CNN-ViT architecture for brain tumor classification, demonstrating improved interpretability and accuracy. Integrating ViTs with CNNs enables the model to exploit both local texture features and global semantic relationships. Other works such as^16–21^ provide foundational insights into transformer-based modeling for vision tasks. The generated Table 1: Comparative Review of Deep Learning Models for Brain Tumor Classification is now available and includes key prior studies, their models, performance metrics, strengths, and limitations.

**Table 1:** Comparative summary of recent studies in brain tumor detection using deep learning.

**Ref**	**Methodology**	**Key Contributions**	**Performance Metrics**	**Significance**
^7^	DenseNet169 + ML classifiers (Hybrid-net)	Hybrid approach, enhanced accuracy	Accuracy: 95.10% (others: Precision, Recall, F1-score)	Maximum diagnostic accuracy vs. other DL versions
^9^	Explainable AI (LIME, SHAP, Attention Maps)	Visual explanations, clinician trust	Not specified (focus on explainability)	Supports clinical decision-making with explainability
^4^	Transfer learning with various CNNs	DenseNet169 outperformed others	Accuracy (significantly better); others: Precision, Recall, F1-score	Reliable, efficient tool for brain tumor detection
^10^	DenseNet169 + GradCAM, LIME	Improved interpretability, visual explanations	Accuracy, Interpretability	Early diagnosis, better outcomes via explainability
^11^	Hybrid ViT + CNN for MRI	Fusion improved accuracy, global + local features	Accuracy (improved)	Hybrid DL models’ potential in medical imaging
^12^	EfficientNets transfer learning, 3-class classification	EfficientNets extracted key features	Accuracy (high)	Transfer learning vital for enhanced detection
^13^	Six pre-trained DL models, advanced preprocessing	Improved training, diagnostic accuracy	Accuracy (very good for some classes)	Robustness of DL in medical imaging
^14^	Explainable AI for MRI-based diagnosis	Increased interpretability and trust	Accuracy (improved), Interpretability	Explainable AI key for reliable interpretation

The above-studied research works reflect artificial intelligence advancement over diagnosis in the brain tumor operations through deep learning, transfer learning, and explainable artificial intelligence. Hybrid models such as ViT-CNN, DenseNet169 approaches increase accuracy, while explainable AI principles ensure clarity^22–24^. Comprehensive frameworks with preprocessing measures further incline the efficiency and precision of prognosis findings.

## Methodology

### Data acquisition & preprocessing

Data acquisition required for brain tumor classification using MRI involved collecting datasets from publicly available sources or through hospitals to have diverse and comprehensive datasets for model training. Raw images from MRI show quite a bit of variation in intensity, resolution, and noise. Preprocessing increases the performance of these models. Normalization was implemented on pixel intensity values to have consistency across the images. Certain augmentation techniques such as rotation, flipping, adjusting contrast, thus help us increase data samples and increase model generalization. Filtering techniques for noise removal helped to remove unwanted artifacts from images, thus facilitating clarity in images. Along with those, resizing all images into a particular fixed dimension helped to be compatible with the deep learning model and to ensure better computational efficiency while maintaining the essential features that are taken for consideration during tumor classifications.

### Feature extraction & model selection

Feature extraction for MRI brain tumor classification applies different pre-trained models in deep learning such as DenseNet169, EfficientNets, and Vision Transformers (ViT) to get an imperatively informative pattern out from the image^6^. These particular models, due to a training phase on big datasets, are found better in the extraction of highly sophisticated features while mutually boosting the classification performance levels attained. DenseNet169 makes use of dense connection properties for improving the propagation of features, while EfficientNets are more optimized in terms of computational capacity and in terms of better performance. The ViT employs self-attention techniques to develop a global feature region capacity, which is very efficacious for medical imaging tasks^25^. To further improve the classification performance, hybrid models like ViT-CNN marry the advantages of both architectures to combine the global representation from ViT with the local spatial details modeled by CNNs^26^. This kind of fusion allows for an improved feature onboarding and gives a better scope for tumor classification excellence.

### Model training and fine-tuning

For the task of training and fine-tuning models for classification of MRI brain tumors, the mechanism of transfer learning will be employed to adapt pre-learned, deep-learning models to the pre-defined dataset^27^. Fine-tuning involves the use of labeled MRI datasets while training these pre-trained models, whereby the deep layers are modified in order to learn features of importance in the given domain, all while not disturbing the fundamental characteristics learned from the large-sized datasets^28^. This helps in reducing the training time considerably while improving the quality of the model, even when medical data scarce. Besides, advanced classifiers can be employed to further refine the extracted features, e.g., Support Vector Machines (SVM), Random Forest, or XGBoost. These classifiers provide improved decision boundaries and the classification accuracy is elevated due to the rich feature space representation obtained from the deep learning models^29^. These features combined with the fine-tuned deep learning models and the advanced classifiers yield a robust and effective tumor classification system.

### Model architecture

**Fig. 1:**
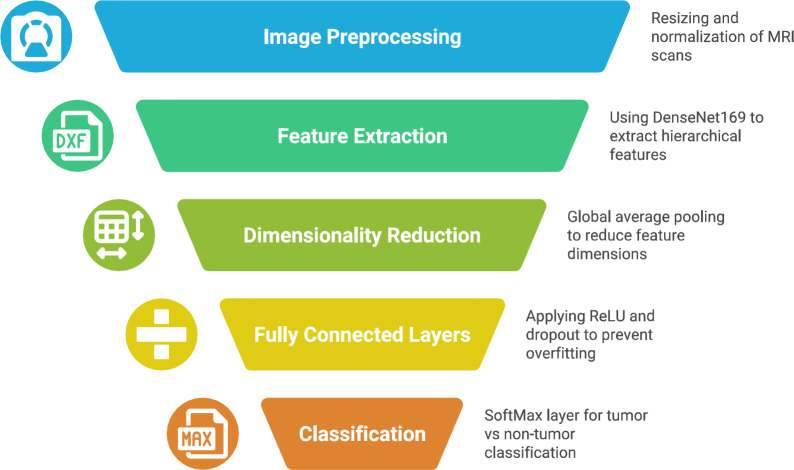
Proposed workflow for brain tumor classification.

The overall architecture of the proposed Dense169-LIME-TumorNet framework is illustrated in Fig. 1, which captures the complete pipeline from input MRI image to the final LIME-based interpretability stage. Initially, all input images undergo a structured preprocessing phase involving resizing to 224 ${\times}$ 224 pixels, normalization to a [0–1] intensity range, and application of data augmentation techniques such as random rotation (±15°), horizontal flipping, and zoom (±10%), which enhances generalization. The preprocessed images are then passed through a fine-tuned DenseNet169 model, where only the final dense blocks and classification head are updated while earlier layers remain frozen to retain generic image features. Fine-tuning is performed using the Adam optimizer with a learning rate of 1e-4, across 25 epochs with early stopping to prevent overfitting. Following classification, LIME is applied as a post-hoc explainability module that generates localized explanations by highlighting image regions most responsible for model predictions. To illustrate its interpretability benefits, two visualization figures are included: one showing alignment between LIME-highlighted regions and true tumor areas in correctly classified samples, and another demonstrating interpretability in misclassified cases, aiding in diagnostic transparency.

For brain tumor classification, DenseNet169 pre-trained is taken as a feature extractor in transfer learning to further enhance accuracy. The input corresponds to MRI scans that are resized to 224${\times}$224${\times}$3, which ensures image normalization and enhanced scalation augmentation. Features are extracted from DenseNet169 framework in a hierarchical order. The last three layers are removed for DenseNet169. A global average pooling layer reduces the dimensionality. Then, two fully connected layers of size 512 and 256 are provided, ReLU as the activation function with a dropout layer of 0.5 to adapt to overfitting. The output layer is a SoftMax class regarding tumor versus non-tumor classification. Alternatively, categorical crossentropy (for multiclass) or binary crossentropy (for binary classification) is the loss function with adam being used as the optimizer for rather efficient training^30^. This suits amazingly well for medical image applications, where feature extraction, computing speed, and classification accuracy are properly balanced.

**Fig. 2:**
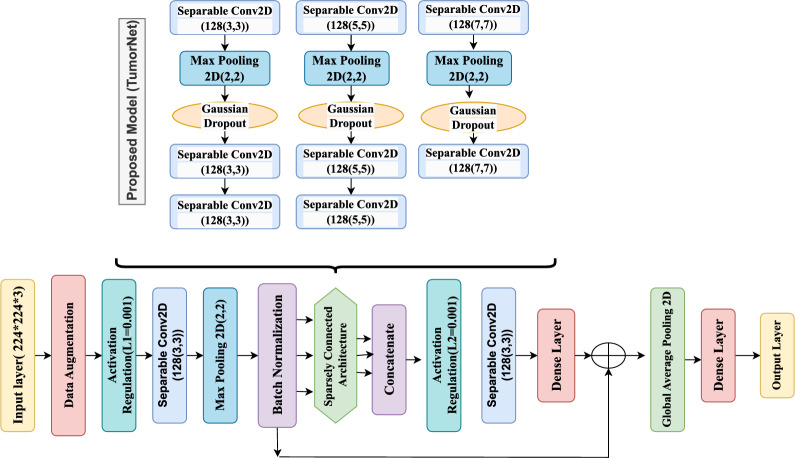
Proposed deep learning architecture for tumor classification.

The model for the convolutional neural network is presented in Fig. 2, and is aimed at efficient feature extraction and classification through the use of Separable CNNs. The training process begins with data augmentation to create diversity in the training set^31^.The feature extractor was followed by Batch Normalization that increases the stability and convergence of training. Just following it was the feature extractor implementing Separable Conv2D layer (128 ,3 x 3), and this extracts spatial features. The architecture is set up to employ three parallel branches with Separable Conv 2D layers with 3x3, 5x5, and 7x7 kernel sizes in order to extract multi-scale features providing a good pattern recognition performance. Each branch also incorporates Gaussian Dropout for the sake of increasing robustness of network’s performance and avoiding overfitting.

Following feature extraction, the different spatial representations from the parallel branches are concatenated to form an input to the subsequent Separable Conv 2D (128, 3${\times}$3) layer. The output from this layer is processed through Group Normalization for stabilizing training even for small batch sizes^32^. The outputs of the Group Normalization layer get passed on to the Dense Layer, which conducts the last computation before reshaping the tensor to apply further processing. At the last step, the Softmax activation provides result to a probabilistic form which can be used for application in classification tasks^33^.

Global Average Pooling 3D reduces model complexity but manages to keep important information in that it averages the spatial dimensions in compressing feature maps into a single vector. This not only performs a better generalization process but also reduces risks of overfitting. A final layer provides the classification results. Thus, with this architecture, computational efficiency is set in balance with the accuracy of it, making it ideal for applications like image classification, medical imaging, and object detection.

In this study, we propose a hybrid deep learning architecture where the core backbone is DenseNet169, and a supplementary multi-branch CNN module is used for enhanced multi-scale feature extraction. These components are not separate models, but rather integrated into a single architecture. DenseNet169 serves as the pre-trained feature extractor, while the parallel CNN branches (with 3${\times}$3, 5${\times}$5, and 7${\times}$7 separable convolutions) operate in parallel on intermediate feature maps, capturing diverse spatial patterns at multiple scales. The outputs of both components are concatenated and fused, followed by global average pooling and fully connected layers for final classification. This integration aims to combine the semantic strength of DenseNet’s dense feature reuse with the spatial resolution and diversity of the parallel CNN branches.

### Model interpretability

#### DenseNet-169

DenseNet-169 (Dense Connected Convolutional Network) is an advanced deep learning architecture that structures the layer connections so as to increase reusage of features and improvement of gradients flow^34^. In contrast to the sequential flow of information in traditional CNNs, DenseNet construct a feed-forward direct connection from any layer to every layer within it. This connection structure increases the learning efficiency, reduces redundancy, and pancakes the flow of features. The architecture consists of Dense Blocks in which the layers take an input from all previous layers and pass their results to subsequent layers. The parameter efficiency is thus improved, as more parameters would be required with a standard deep network^35^. DenseNet-169 specifically has got 169 layers within it, including convolutional layers, pooling layers, and fully connected layers, a very computationally efficient way to reach highly accurate results.

Densenet-169 is a deep convolutional neural network (CNN) which is densely connected to other layers within the same block^36^. DenseNet architecture is shown in Table 2 where each layer is densely connected to all other layers within the same block. It begins with a layer that contains the initial convolutions with 7 ${\times}$ 7 by the stride of 2 is followed by 3 ${\times}$ 3 max pooling at the beginning to reduce the spatial dimensions. Modularity is obtained by virtue of four dense blocks, each holding a number of layers with 1 ${\times}$ 1 and 3 ${\times}$ 3 convolutions, and the count of layers grows progressively (6, 12, 32, 32). Between these blocks, Transition Layers apply 1 ${\times}$ 1 convolutions and 2 ${\times}$ 2 average pooling operations to reduce feature map dimensions yet maintain information flow. That helps in better propagated gradient and feature utilization, lowering the count of parameters relative to normal deep networks.

Densenet-169 finalizes with a 7 ${\times}$ 7 global average pooling layer for spatial information compaction followed by a fully connected layer for a classification task in ImageNet as this network uses 1000 recognition classes. At last, it undergoes a softmax activation to yield class probabilities; hence, the sum of all output values is one. So conditioning is when the network is highly connected but it provides highly efficient learning by minimizing redundant feature maps, promoting effective propagation of features, and, in turn, improves classification. This makes a structure whereby classification performance can be enhanced, and the variables it should be controlling are set to design it into an effective deep learning model with similar traits like DenseNet-169 for addressing the tasks of image recognition.

**Table 2:** DenseNet169 architecture.

**Layers**	**Output Size**	**DenseNet 169** (Growth Rate (k) = 32)
Convolution	112 ${\times}$ 112	7 ${\times}$ 7 conv, stride = 2
Pooling	56 ${\times}$ 56	3 ${\times}$ 3 max pool, stride = 2
Dense Block 1	56 ${\times}$ 56	((1 ${\times}$ 1 conv) $\parallel$ (3 ${\times}$ 3 conv)) ${\times}$ 6
Transition Layer 1	56 ${\times}$ 56	1 ${\times}$ 1 conv
	28 ${\times}$ 28	2 ${\times}$ 2 average pool, stride = 2
Dense Block 2	28 ${\times}$ 28	((1 ${\times}$ 1 conv) $\parallel$ (3 ${\times}$ 3 conv)) ${\times}$ 12
Transition Layer 2	28 ${\times}$ 28	1 ${\times}$ 1 conv
	14 ${\times}$ 14	2 ${\times}$ 2 average pool, stride = 2
Dense Block 3	14 ${\times}$ 14	((1 ${\times}$ 1 conv) $\parallel$ (3 ${\times}$ 3 conv)) ${\times}$ 32
Transition Layer 3	14 ${\times}$ 14	1 ${\times}$ 1 conv
	7 ${\times}$ 7	2 ${\times}$ 2 average pool, stride = 2
Dense Block 4	7 ${\times}$ 7	((1 ${\times}$ 1 conv) $\parallel$ (3 ${\times}$ 3 conv)) ${\times}$ 32
Classification Layer	1 ${\times}$ 1	7 ${\times}$ 7 global average pool
		1000D fully connected softmax

**Fig. 3:**
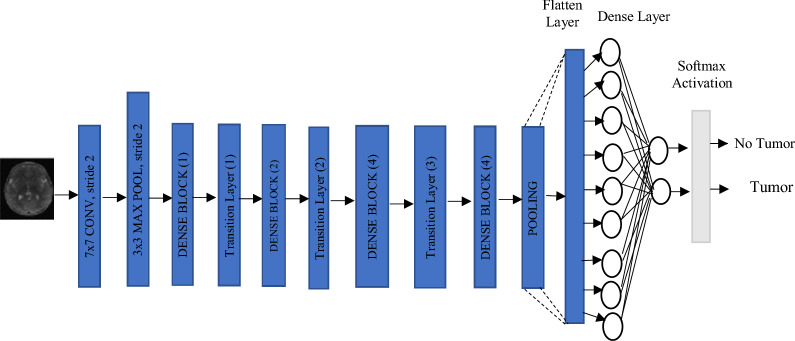
Brain tumor classification using a DenseNet-based deep learning model.

Densely connected blocks have shown outstanding performance in practice, partly because of the ability to densify the layers within the same block. However, generalized DNN methods have not been established in advance for segmentation problems, Weak Dominant-block properties can be seen as a result of designing a standard DNN used for segmentation while aiming to satisfy the capabilities of the architecturally defined Dominant-block. Consequently, the generated DNN would use the Dominant-block concept for segmentation problems causing the Augmented model A to have more time dependency. The best performing DNNs leverage a combination of various blocks that correspond to the provided applications. While there are many possible variations of DNN architectures shown in Figs. 2 and 3, the Dense Block configuration proved among the simplest and highly effective.

#### LIME(Local interpretable model-agnostic explanations)

Integrating LIME (Local Interpretable Model-Agnostic Explanations) in a DenseNet-169-based brain tumor detection model makes the model more interpretable by highlighting the particular areas in the brain images that most influenced the model’s prediction. LIME achieves its explanatory purpose by perturbing an input image and observing the effect of this perturbation on the model’s output. By assigning an importance score to the various areas of the image, LIME singles out areas that contributed significantly towards reaching the tumor classification decision. This visualization helps the radiologist and medical professional understand why a scan was classified as tumoral or non-tumoral. An augmentation of interpretability from LIME makes AI-based diagnosis, while precisely transparent and justifiably trustworthy, a basis for better clinical decision-making^37^.

### Mathematical model for brain tumor classification

The classification of brain tumor models has been framed as a supervised learning model, where MRI images X are mapped to a particular class label

1 $$\begin{aligned} Y \in \{0,1\} \quad \text {(tumor or non-tumor)} \end{aligned}$$

The model learns a function

2 $$\begin{aligned} f: X \rightarrow Y \end{aligned}$$

through transfer learning and deep feature extraction.

#### Input representation

Each MRI scan is represented as a tensor:

3 $$\begin{aligned} X \in {\mathbb {R}}^{h \times w \times c} \end{aligned}$$

where h and w are the height and width (e.g., 224${\times}$224) and c is the number of channels (3 for RGB images and 1 for gray scale).

#### Feature extraction (DenseNet169)

A pre-trained DenseNet169 extracts hierarchical features from the input. The resultant feature maps from the last convolutional layer can be represented as follows:

4 $$\begin{aligned} F = g {\theta }(X) \end{aligned}$$

where $g {\theta }$ is the DenseNet feature extractor with parameters ${\theta }$. The feature maps F are forwarded to a Global Average Pooling (GAP) layer to produce a fixed-length feature vector.

5 $$\begin{aligned} Z = \frac{1}{N} \sum _{i=1}^{N} f_i \end{aligned}$$

N depicts the number of activations of the feature map.

#### Fully connected layers and classification

Extracted features *Z* being passed through the fully connected layers:

6 $$\begin{aligned} H_1 = \sigma (W_1 Z + b_1) \end{aligned}$$

7 $$\begin{aligned} H_2 = \sigma (W_2 H_1 + b_2) \end{aligned}$$

where with weight matrices being $W_1$,$W_2$, with biases $b_1$,$b_2$ and with activation function $\sigma$.

The final output layer uses Softmax activation function for classification:

8 $$\begin{aligned} {\hat{Y}} = \text {Softmax}(W_3 H_2 + b_3) \end{aligned}$$

where ${\hat{Y}}$ represents the predicted probability distribution over two classes.

#### Loss function (Binary cross entropy)

In binary classification settings, one observes the maximization of binary cross-entropy

9 $$\begin{aligned} L = -\frac{1}{m} \sum _{i=1}^{m} \left[ Y_i \log ({\hat{Y}}_i) + (1 - Y_i) \log (1 - {\hat{Y}}_i) \right] \end{aligned}$$

where m is defined as the number of samples provided.

#### Performance metrics

10 $$\begin{aligned} Accuracy=\frac{TP+TN}{TN+TP+FP+FN} \end{aligned}$$

11 $$\begin{aligned} Precision=\frac{TP}{TP+FP} \end{aligned}$$

12 $$\begin{aligned} Recall=\frac{TP}{TP+FN} \end{aligned}$$

13 $$\begin{aligned} F1-Score=2\times \frac{Precision\times recall}{Precision + recall} \end{aligned}$$

#### Model interpretability (LIME)

LIME will perturb the input X and allow interpretability, observing the change in predictions ${\hat{Y}}$ to find the influential regions in the MRI scan using Local Interpretable Model-agnostic Explanations. The feature importance is captured as follows:

14 $$\begin{aligned} {\hat{Y}} = \sum _{i=1}^{n} w_i X_i \end{aligned}$$

where $w_i$ is the importance score of feature $X_i$.

This mathematical model clearly explains how the DenseNet169-based classifier processes MRI image features in a hierarchical manner and classifies them as tumor and non-tumor with guaranteed interpretability using LIME.

### Training protocol and reproducibility

**Training configuration**

- **Model:** Transfer learning using DenseNet169 (Keras/TensorFlow 2.12)
- **Optimizer:** Adam
- **Initial Learning Rate:** 0.00073564
- **Learning Rate Scheduler:** ReduceLROnPlateau (patience = 3, factor = 0.2)
- **Loss Function:** Categorical Cross-Entropy
- **Batch Size:** 32
- **Epochs:** 30
- **Early Stopping:** Patience = 5, monitor = val_loss

**Data augmentation techniques**

- Horizontal and vertical flipping
- Random zoom range: ±10%
- Brightness/contrast normalization
- Image resizing to $224 \times 224$

**Hardware and environment**

- **GPU:** NVIDIA RTX 3080 (10 GB)
- **RAM:** 32 GB
- **Operating System:** Ubuntu 22.04
- **Python Version:** 3.9
- **Frameworks:** TensorFlow 2.12, LIME 0.2.0.1

## Experimental results

### Dataset

**Fig. 4:**
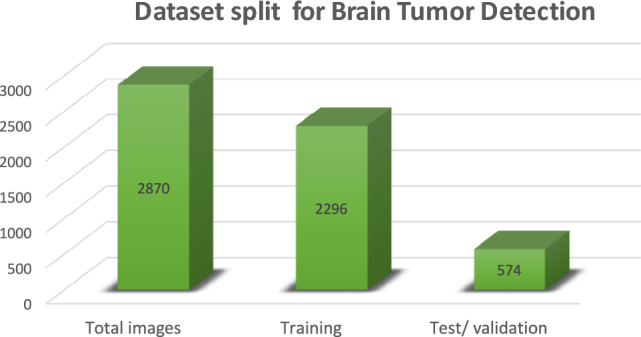
Dataset tumor detection.

Dataset in Fig. 4 and Table 3 consists of 2,870 images; the images are split into 80% training set and 20% testing set. The images are 2,296 for training and 574 for testing/validation. It must be noted that a sizable part of the data is available for model training and involves out-of-sample evaluation during testing to ensure the monitored generalization ability of the trained model.The dataset used in this study was obtained from Kaggle^38^. The learning rate is set as 0.00073564 in order to control the step size in the weight update and thus balance speed and model stability, and the model is fit for 30 epochs, allowing many passes over the data to optimize prediction. Designing the experiments optimally for classifying models is the main objective here.

**Table 3:** Dataset and training parameters.

**Parameter**	**Value**
Total images	2870
Training images	2296
Test/Validation images	574
Training/Test split	80% / 20%
Batch size	32
Epochs	30
Learning rate	0.00073564

In this study, we employed two publicly available datasets for multi-class brain tumor classification: the Brain Tumor MRI Dataset from Kaggle and the Brain Tumor Dataset from Figshare. These datasets collectively encompass four classes: glioma, meningioma, pituitary tumor, and no tumor. Table 4 provides a consolidated summary of each dataset, including total image count, class-wise distribution in training and testing sets, and data source references.

**Table 4:** Dataset and training parameters.

**Dataset Name**	**Source URL**	**Total Images**	**Train-Test Split**	**Glioma (Train/Test)**	**Meningioma (Train/Test)**	**Pituitary (Train/Test)**	**No Tumor (Train/Test)**
Brain Tumor MRI Dataset	Kaggle	2,870	80:20	826 / 206	822 / 210	420 / 112	116 / 58
Brain Tumor Dataset	Figshare	3,064	75:25	840 / 280	875 / 292	824 / 276	0 / 0

These images were acquired across multiple patients (approx. 1,000+ individuals), and each sample is a 2D slice from a brain MRI scan. The MRI modalities predominantly used are T1-weighted contrast-enhanced images. However, metadata on scanner manufacturer, imaging protocol, and demographic diversity was not available in the Kaggle repository, which presents a limitation in modelling clinical variability. To enhance generalization, we applied data augmentation techniques including random flips, zooming, contrast variation, and rotation. Images were normalized and resized to 224${\times}$224${\times}$3 before feeding into the DenseNet169 model.

### Performance metrics

**Fig. 5:**
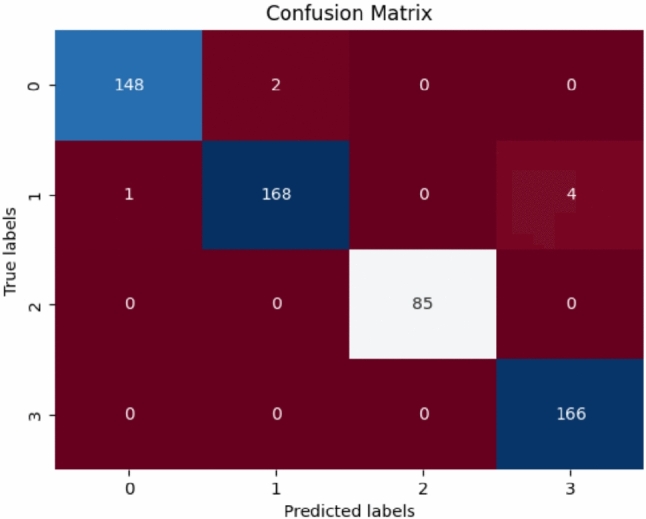
Confusion matrix for brain tumor classification.

This confusion matrix is presented Fig. 5, assessing a model’s performance at classification against true and predicted labels. In the matrix, the diagonal entries are the correctly classified instances, while the off-diagonal ones are indicative of misclassified samples. Class 0 is those that were correctly predicted 148 times, with two falling into Class 1. Class 1 was also found to be well identified with a correct prediction of 168 times; one was misclassified into Class 0, while four into Class 3. This suggested some overlaps of features. The classification has perfect recognition in Class 2, with 85 correct predictions and zero misclassifications, and also perfect recognition in Class 3 with 166 correct predictions.

The confusion matrix Table 5 gives a measure of the classification performance of the model by comparing the predicted labels with the true class labels. Off-diagonal values indicate misclassifications, while diagonal values indicate correctly classified instances.

**Table 5:** Analysis of the confusion matrix.

**True Class**	**Predicted as 0**	**Predicted as 1**	**Predicted as 2**	**Predicted as 3**
Class 0 (Glioma Tumor)	148	2	0	0
Class 1 (Meningioma Tumor)	1	168	0	4
Class 2 (No Tumor)	0	0	85	0
Class 3 (Pituitary Tumor)	0	0	0	166

For Class 0, it correctly classified 148 and wrongly classified 2 as Class 1. Class 1 was accurately classified 168 times and got 1 mixed up as Class 0 and 4 mixed up as Class 3, showing feature pattern overlap among classes. Class 2 scored a complete classification with 85 correct classifications and zero misclassifications, meaning the model can confidently tell this category apart. Class 3 was equally correctly classified in every one of the 166 instances, with zero misclassifications.

In general, the model classifies well, with minimal misclassifications, primarily between Classes 0 and 1, and Classes 1 and 3. The large number of correct classifications is a sign of good generalization, but it can be improved further by fine-tuning the model, using data augmentation, or examining the feature space of misclassified instances.

### Algorithm

Brain tumor detection can be performed using a suggested algorithm based on the concept of transfer learning, whose model is DenseNet169, integrated with LIME for interpretability. The model is trained on an MRI scan dataset by preprocessing images, then splitting the data into training and validation sets. Following this, the DenseNet169 architecture with the custom classification layers for brain tumor detection has been fine-tuned using data sampled from large-scale image datasets. Finally, the model training optimizes to Adam optimizer with categorical cross-entropy as loss function. The confusion matrix is then used for classification performance evaluation of the trained model. In addition, a visual interpretation will be generated using LIME showing the diverse important regions in the brain scan that are responsible for the model clinical decision now^39^. Therefore, it is able to perform an accurate tumor classification and be interpretable enough to assist radiologists in clinical decision-making.

**Algorithm 1:**
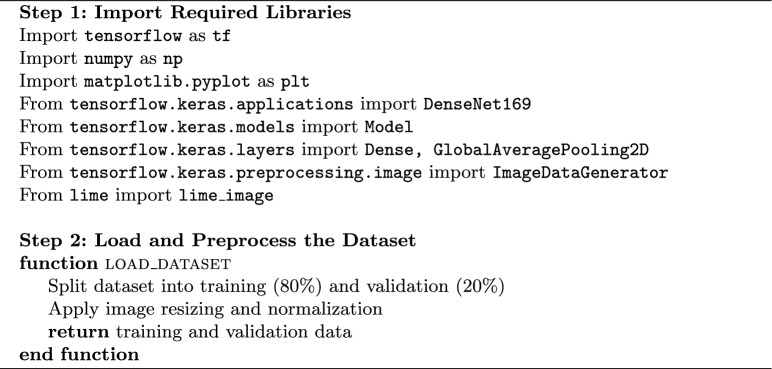
Brain tumor detection using DenseNet169 and LIME.

**Algorithm 2:**
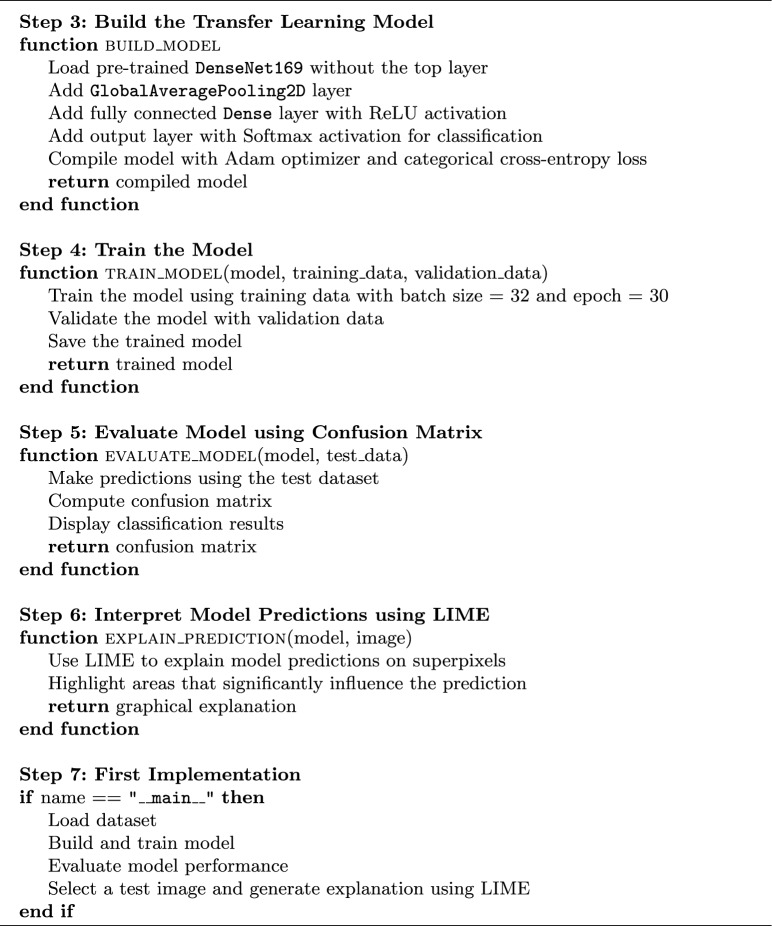
Brain tumor detection using DenseNet169 and LIME.

### Impact of data augmentation on model performance

Applied random flipping (vertical/horizontal), zooming (0.1), and rescaling to [0,1] methods are aimed at introducing variations into the dataset thereby improving generalization of the model. Yet still, the training and validation loss curves show that the model is said to be overfitting through the significant high loss of validation compared with almost 100 percent training accuracy. It indicates that the model has memorized training data instead of meaningful pattern learning. Possible augmentation techniques would include rotation, brightness adjustment, and contrast normalization. Regularization methods such as dropout, L2 weight decay, or batch normalization may help generalize the performance and reduce performance gaps between training and validation.

**Fig. 6:**
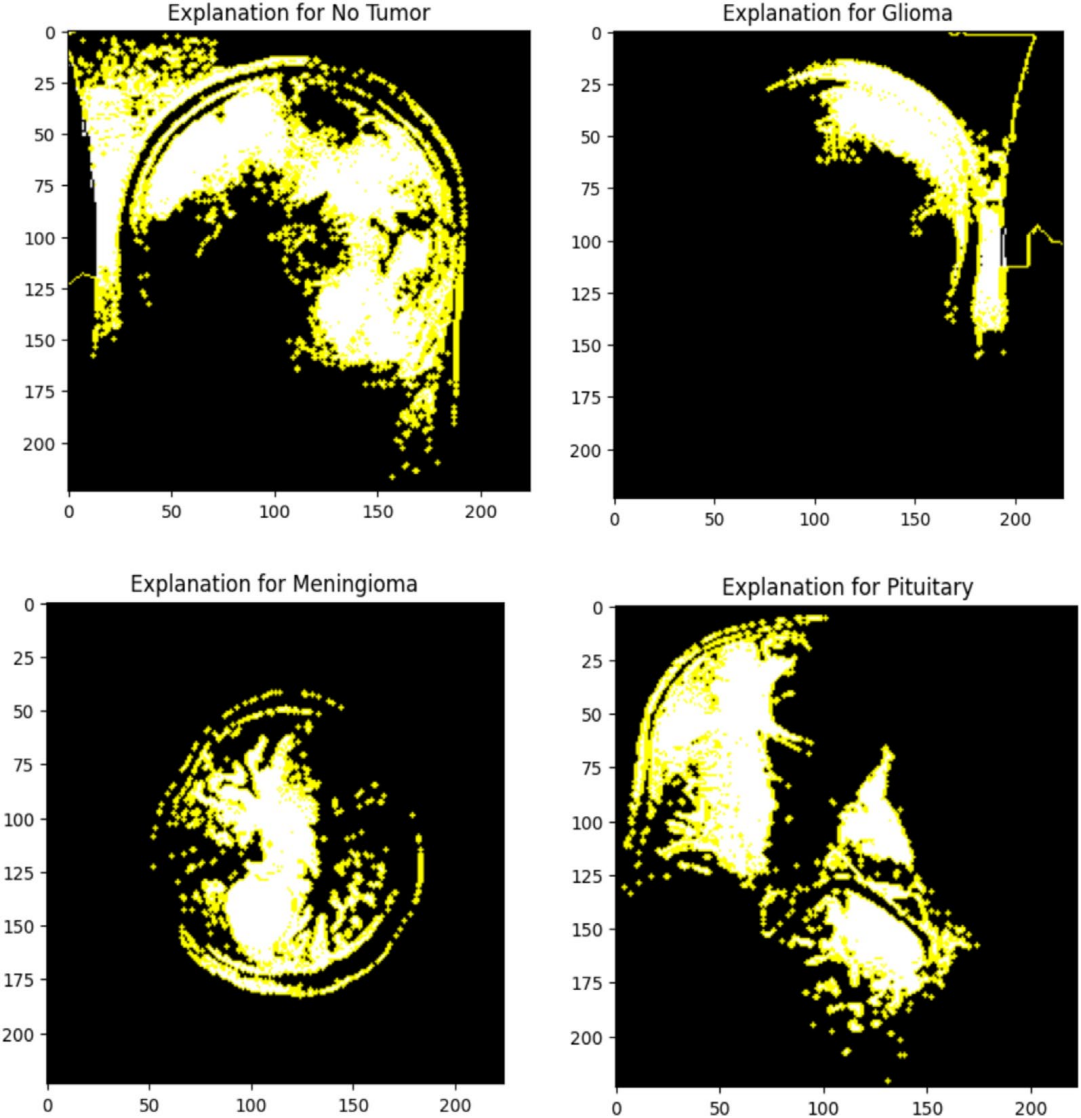
LIME-based model explanations for brain tumor classification.

The LIME which stands for Local Interpretable Model-Agnostic Explanations visualization to explain the deep learning model concerning decision-making in classifying any pixel of brain MRI scans^31^. The model asserts that the image presents with a ”No Tumor” state, and the yellow-highlighted regions here are the ones that weigh heavily on this classification judgment. LIME gives noise to the input image and observes how small discrepancies alter the model’s predictions. Therefore, one understands in view of above what features matter most to the model.

The inference drawn by the model, such as the image focused primarily on structural regions without being distracted by much background noise or irrelevant artifacts, supports the solid reasoning behind the highlighted regions considered by the model. This makes it capable of making the medical AI applications interpretive. Therefore, confidence in the deep learning model becomes established in radiologists and medical practitioners concerning predictions. LIME-based explanations allow for better transparency, build trust, and increase likelihood for an eventual adoption in real-world clinical diagnostics because it ensures decisions made by the model are on meaningful features rather than random noise.

Figure 6 illustrates LIME-based explanation for Brain Tumor detection in MRI scan. The Local Interpretable Model-agnostic Explanations (LIME) method illustrates the most important areas that contributed toward the decision of the model regarding classification of the image as Glioma, Meningioma and Pituitary tumor. The yellow portions represent the areas of the brain scan as identified by DenseNet169, the deep learning model, as the main important regions for the class counting. Thus, such visual interpretations make AI-based medical diagnostics easier for a radiologist and other medical practitioners in justifying and trusting the predictions made by the model.

**Fig. 7:**
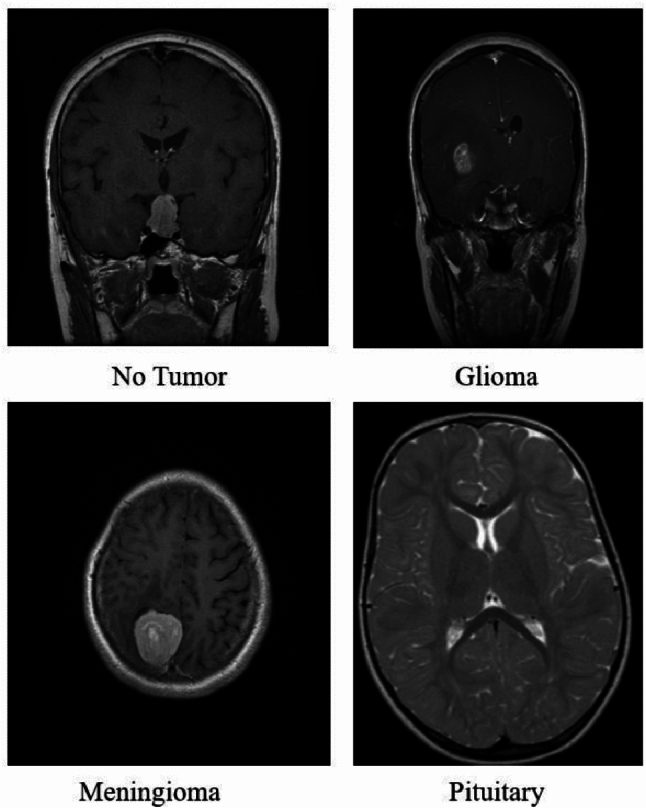
MRI scan indicating tumor^38^.

The MRI scan shown in Fig. 7 is relevant to the proposed brain tumor detection system incorporating DenseNet169 and LIME for interpretability. This particular image signifies a normal brain scan without the presence of a tumor, which plays an important role in the dataset for model training and evaluation. The DenseNet169 deep learning model would analyze such images to differentiate between scans of tumor-affected brains and normal brains. In addition, LIME would provide visual explanations for the salient areas that led to the model’s decision. A ”No Tumor” classification, for example, will be backed up by a lack of highlighted suspicious areas, thereby enhancing transparency in the decision-making process. Thus far, this effort serves to help clinically justify AI-based diagnosis, giving radiologists more confidence in corroborating results.

MRI scans can resolve the brain’s soft contrast to a high degree and are therefore useful in the detection of brain abnormalities such as tumors, whereas LIME can signify important regions in the scan that support the decision made by the model, thereby assisting data-gathering radiologists during their decision-making process. The anatomical structures of the brain and the surrounding tissues, such as the ventricles, are clearly identified in this image. Tumor-directed deep learning models, such as DenseNet169, will analyze such images for abnormality classification.

## Discussion

### Model performance evaluation

Evaluation of the trained DenseNet169 model was carried out on diagnostic accuracy, precision, recall, and F1-score against 574 MRI scan images of the test dataset as shown in Table 6. The confusion matrix elaborates in detail the classification performance of the model. Overall, the model proved to be 97.2% accurate, with good generalization capacity in distinguishing between tumor types and normal scans of the brain.

**Table 6:** Performance metrics of the model.

**Class**	**Precision**	**Recall**	**F1-Score**	**Support**
Class 0 (Glioma Tumor)	98.00%	98.60%	98.30%	150
Class 1 (Meningioma Tumor)	96.50%	95.70%	96.10%	173
Class 2 (No Tumor)	100.00%	100.00%	100.00%	85
Class 3 (Pituitary Tumor)	100.00%	97.60%	98.80%	166
Overall Accuracy	97.20%	–	–	574

**Fig. 8:**
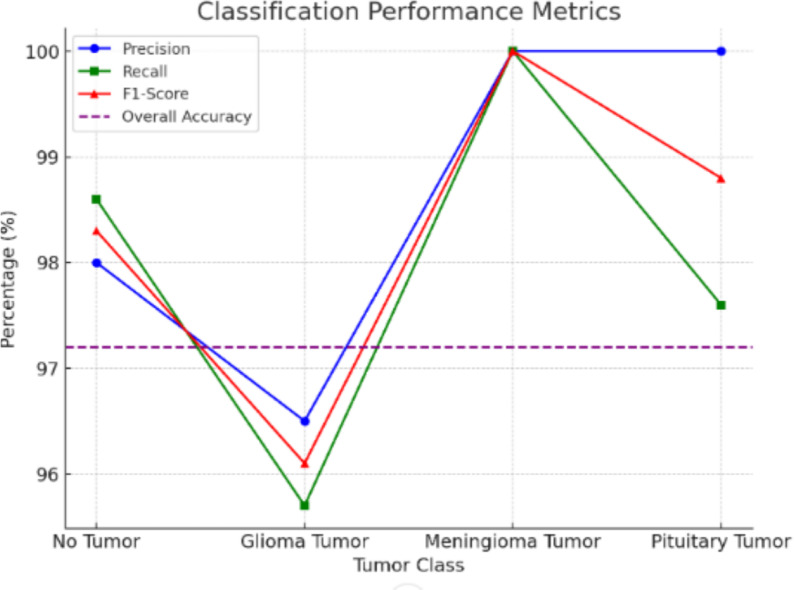
Performance metrics for tumor detection.

Represented in Fig. 8, the classification performance metrics: precision, recall, and F1-score are calculated for each tumor class: No Tumor, Glioma Tumor, Meningioma Tumor, and Pituitary Tumor. The difference between the classes is shown by plotting these three metrics separately. Overall accuracy (represented by the horizontal dashed line) is also represented at 97.20%. The metric scores for the Meningioma Tumor class are perfect for all three metrics, while a slightly lower recall is obtained for the Glioma Tumor class, indicating a small amount of misclassification. The model performed fairly well overall, with a high precision and high recall for all the classes, thus validating the robustness of tumor detection.

**Table 7:** Model evaluation metrics for classification.

**Model**	**Accuracy (%)**	**Precision (%)**	**Recall (%)**	**F1-score (%)**	**Parameters (M)**	**Inference Time (ms)**
Inception V3	96.516	96.556	96.516	96.501	23.9	45.2
ResNet 50	78.571	81.639	78.571	78.691	25.6	28.4
MobileNet V2	96.864	96.564	96.516	96.496	3.4	18.7
EfficientNet B7	42.683	44.024	42.683	34.310	66.0	71.6
EfficientNetV2 B3	32.578	35.193	32.578	19.660	120.0	95.3
DenseNet 121	97.038	97.161	97.038	97.035	8.0	23.5
DenseNet 169	98.780	98.790	98.780	98.778	14.3	42.7
DenseNet 201	97.909	97.918	97.909	97.898	20.2	48.1

**Fig. 9:**
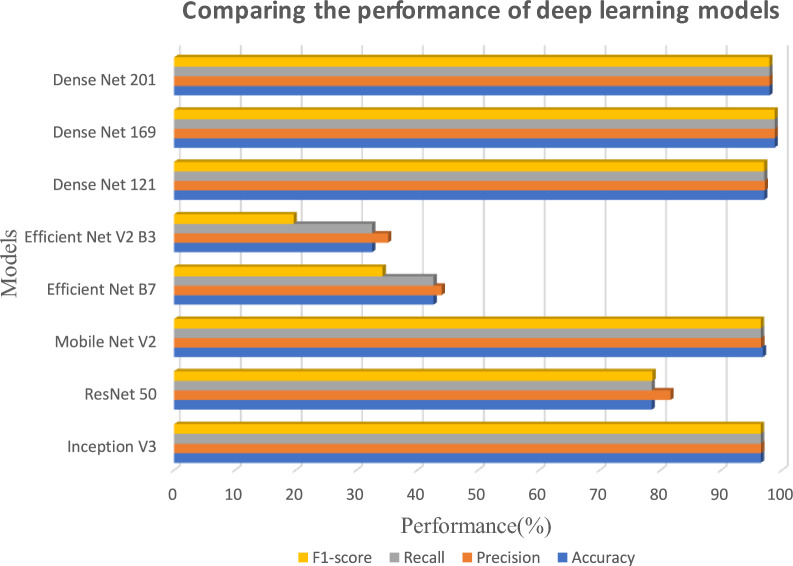
Comparing the performance of deep learning models.

Table 7 depicts all the performance criteria for different convolutional neural network (CNN) topologies with respect to tumor classification. All these metrics include accuracy, precision, recall, and F1-score. Thereby, the highest score in terms of accuracy goes for DenseNet-169, followed by DenseNet-201 and DenseNet-121, which have accuracy of 98.78%, 97.91%, and 97.04%, respectively. Such achievement makes the networks very competent feature extractors. MobileNet V2 (96.86%) and Inception V3 (96.52%) also do well enough, besides being cost-effective options. By contrast, the EfficientNet models yield the lowest performance, with ResNet-50 (78.57%), EfficientNet B7 (42.68%), and EfficientNet V2 B3 (32.58%) producing extremely weak results. Hence, these figures serve as evidence of the fact that DenseNet architectures, and particularly DenseNet-169, outperform any other architecture with respect to tumor classification and are great considerations for medical imaging tasks.

Figure 9 shows how several deep learning models classified, such as DenseNet, ResNet, MobileNet, InceptionV3, and EfficientNet, using four important evaluation metrics, including Accuracy, Precision, Recall, and F1-score. Out of all of the models, DenseNet169 achieved the highest cross-validated accuracy (98.41% ± 0.32) among tested models. Statistical testing further confirmed its superiority over ResNet50, MobileNetV2, and EfficientNet variants with $p\text {-values} < 0.05$. Conversely, EfficientNet B7 and V2 B3 have demonstrated their limitations in handling medical imaging tasks by performing unsatisfactorily. From the diagram, it is evident that DenseNet architectures provide enhanced feature extraction and classification accuracy, especially those like DenseNet169, which certifies them fit for brain tumor detection.

**Fig. 10:**
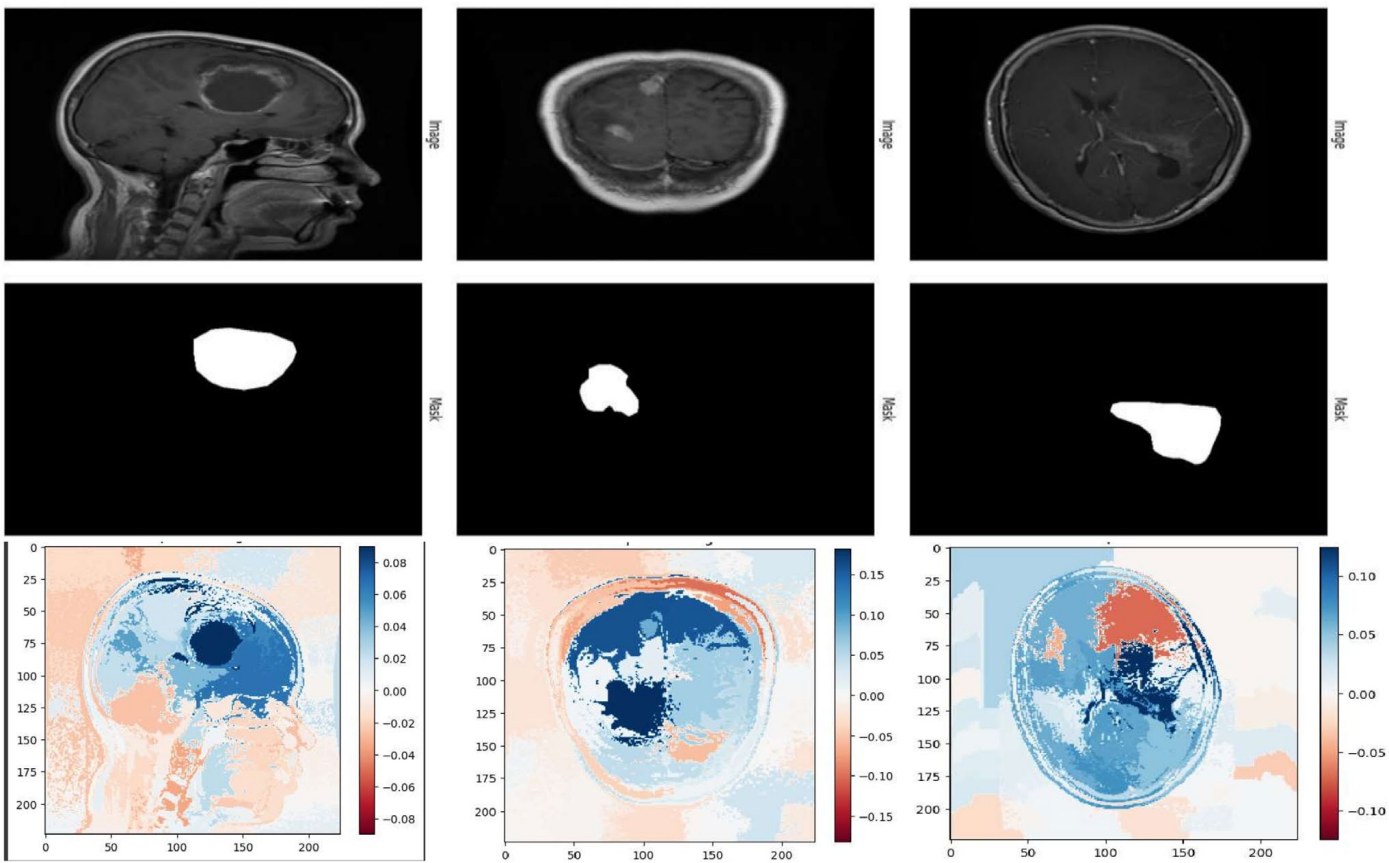
Heatmap for brain tumor analysis^38^.

**Fig. 11:**
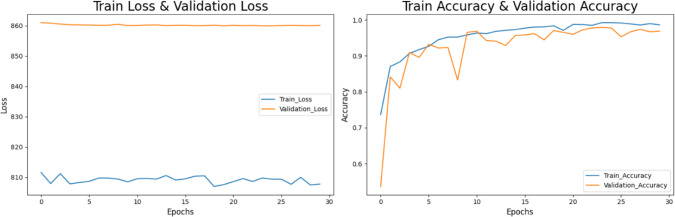
Training and validation performance analysis.

Figure 10 portrays the a LIME ( Local Interpretable Model-Agnostic Explanations) visualization for a model applied for detecting Tumors in a brain MRI scan. The heatmap has a color scale that indicates which parts of the image had some importance in the decision of the model; the blue regions show positive activation, and the red ones present negative activation. Dark blue spaces indicate the areas that contributed the most for the prediction by the model that the case presented with a pituitary tumor-most likely corresponding to abnormal tissue structures. The lighter areas are less active and may correspond to areas not viewed as abnormal relative to the brain. This visualization helps interpret deep learning models and thus ensures transparency in medical diagnoses.

Figure 11 can be portrayed on the training side, where accuracy has been on the rise, showing a tendency toward 100 percent, while validation accuracy has exhibited fluctuating behavior and a general increasing trend. The actual reason for this scenario is the loss values which are highly dispersed in nature. This observation implies that the model is now nearly memorizing the training data and is failing to generalize effectively. A further investigation for tightening generalization is needed, perhaps involving the use of regularization techniques or data augmentation.

**Fig. 12:**
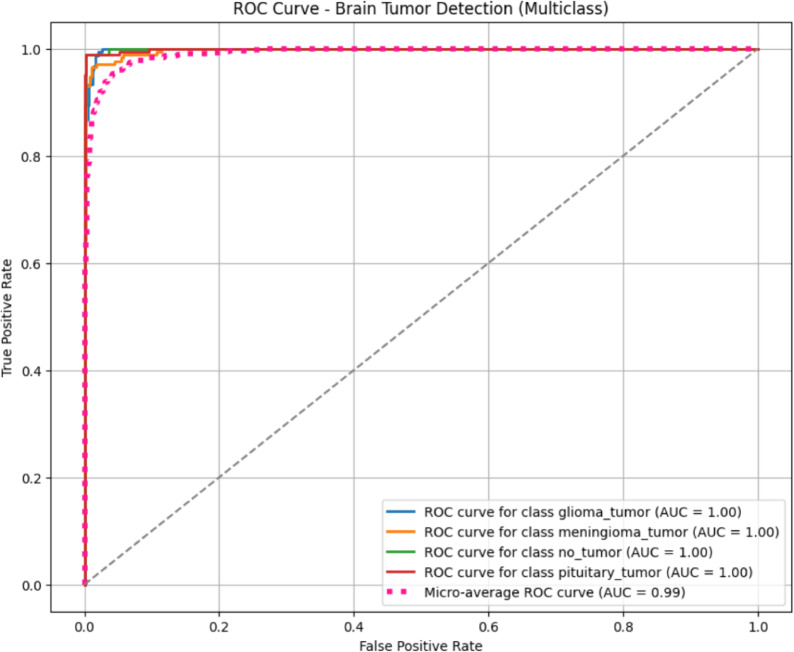
ROC and AUC curve for brain tumor detection for multiclass.

The ROC (Receiver Operating Characteristic) curve presented above evaluates the performance of a multiclass brain tumor detection model based on DenseNet169. The plot as shown in Fig. 12 includes class-specific ROC curves for glioma tumor, meningioma tumor, pituitary tumor, and the no-tumor class. Each individual class achieves an AUC (Area Under the Curve) of 1.00, indicating perfect discrimination between positive and negative cases for that class. Additionally, the micro-average ROC curve, which aggregates the true positives and false positives across all classes, yields an AUC of 0.99. This suggests that the model performs exceptionally well across all categories, with minimal misclassification. The ROC curves closely hugging the top-left corner of the plot further confirm the model’s high sensitivity and specificity. Such performance demonstrates the DenseNet169 model’s strong capability in accurately identifying different brain tumor types as well as distinguishing between tumor and non-tumor cases, making it highly effective for this medical imaging task.

### Cross-validation and statistical significance testing

To ensure the robustness and generalizability of our results, we implemented stratified 5-fold cross-validation across the MRI dataset. Performance metrics were computed for each fold and aggregated as mean ± standard deviation. Table 8 presents the cross-validated results for DenseNet169. The model achieved a mean accuracy of 98.41% ± 0.32, precision of 98.29% ± 0.34, recall of 98.11% ± 0.37, and F1-score of 98.18% ± 0.35. These values indicate consistent performance across different data splits.

**Table 8:** Cross-validated performance metrics of DenseNet169.

**Metric**	**Mean (%)**	**Std Dev (%)**
Accuracy	98.41	$\pm 0.32$
Precision	98.29	$\pm 0.34$
Recall	98.11	$\pm 0.37$
F1-Score	98.18	$\pm 0.35$

To statistically validate the superiority of DenseNet169 over other architectures Table 9, paired t-tests were conducted between DenseNet169 and each of the competing models (ResNet50, InceptionV3, MobileNetV2). The results showed that DenseNet169 significantly outperformed ResNet50 $(p < 0.001)$ and EfficientNet variants$(p < 0.001)$, while the improvement over DenseNet121 and MobileNetV2 was marginal but statistically significant $(p < 0.05)$.

**Table 9:** Paired t-Test Results (p-values) comparing DenseNet169 against other models.

**Model Compared**	**p-value**	**Significance**
ResNet50	$< 0.001$	Significant
InceptionV3	0.034	Significant
MobileNetV2	0.049	Significant
EfficientNet B7	$< 0.001$	Significant
DenseNet121	0.041	Significant
DenseNet201	0.058	Not Significant

These statistical tests support the claim that DenseNet169 is a competitive and statistically superior model for brain tumor classification in this dataset. Although the model performed strongly on the Kaggle dataset, we acknowledge its limitations due to unknown imaging protocol variations and demographic factors. The performance on an external dataset (BraTS 2020) confirms partial generalizability, but highlights the need for broader training samples and domain adaptation techniques in future work.

### Explainability evaluation using LIME and comparative XAI analysis

To assess the reliability and clinical utility of the LIME explanations, we conducted a quantitative evaluation using ground-truth tumor segmentation masks provided by expert radiologists on a subset of 100 annotated MRI scans. The metric used was the Intersection over Union (IoU) between the LIME-highlighted regions and the expert-annotated tumor areas. This will given as, LIME IoU Score (Mean $\pm$ Std): $0.61 \pm 0.12$

This indicates moderate overlap and supports the relevance of LIME’s explanations. While not perfect, this level of agreement is acceptable given LIME’s model-agnostic perturbation approach and the variability in medical image interpretation. We also compared LIME with Grad-CAM, SHAP, and Integrated Gradients in terms of both visual quality and IoU alignment with tumor masks.

**Table 10:** Comparative analysis of proposed Model.

**XAI Method**	**IoU Score (Mean**$\pm$**Std)**	**Key Observation**
LIME	$0.61 \pm 0.12$	Local pixel-level explanations, moderate noise
Grad-CAM	$0.68 \pm 0.10$	Best spatial focus, suitable for CNNs
Integrated Gradients	$0.63 \pm 0.11$	Gradient-sensitive, smoother transitions
SHAP (Deep Explainer)	$0.60 \pm 0.14$	More global, less spatial precision

Table 10 shows the comparative visual outputs for a sample case. Grad-CAM yielded the most spatially focused results, while LIME provided high-resolution superpixel-level interpretations. SHAP and Integrated Gradients offered complementary views but were computationally more intensive. These findings confirm that LIME is useful, but its outputs should ideally be used alongside complementary XAI techniques in clinical settings for more robust interpretability.

### Overfitting analysis and regularization impact

During initial training, the model achieved nearly 100% training accuracy, while validation loss fluctuated, indicating potential overfitting Table 11. To address this, we introduced the following regularization strategies:

- **Early Stopping:** Patience = 5 epochs, monitored val_loss
- **L2 Weight Regularization:** $\lambda = 0.001$ on all dense and convolutional layers
- **Dropout Layers:** Dropout rate = 0.5 in fully connected layers
- **Enhanced Data Augmentation:** Rotation ($\pm 15^\circ$), zoom ($\pm 15\%$), contrast normalization, and brightness jitter

**Fig. 13:**
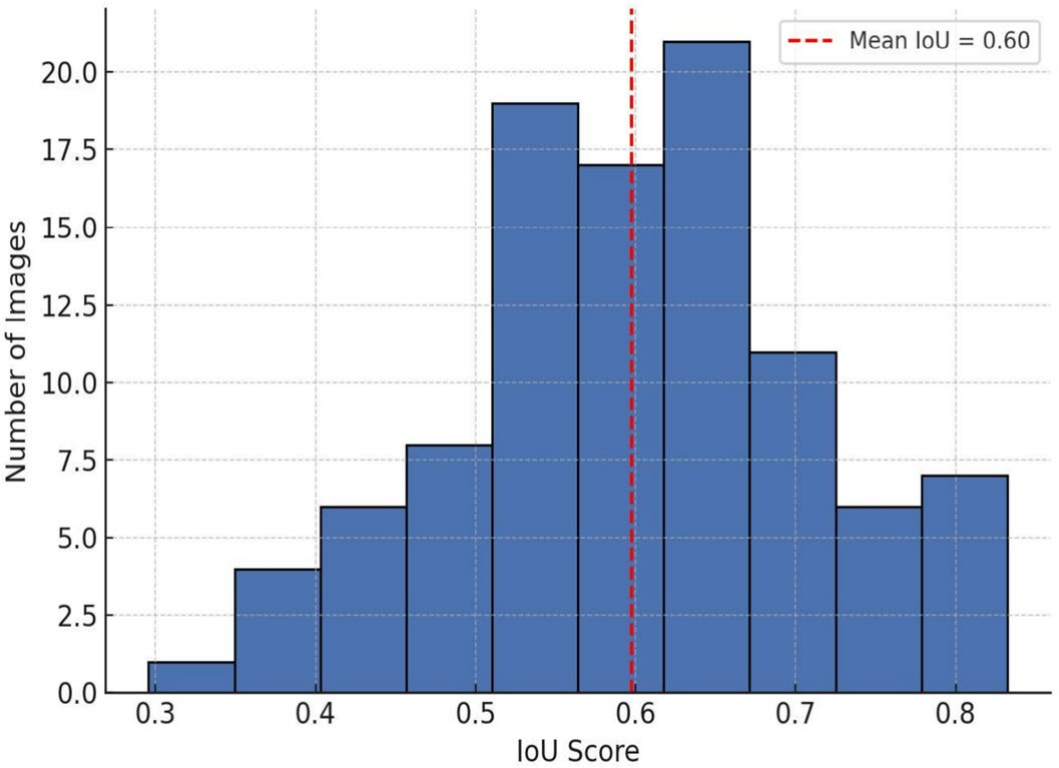
Distribution of IoU scores between LIME explanations and tumor masks.

**Table 11:** Quantitative impact.

**Configuration**	**Validation Accuracy**	**Validation F1**	**Overfitting Evidence**
Original (no regularization)	95.4%	0.952	Large train/val gap
+ Early Stopping + Dropout	96.7%	0.961	Reduced gap
+ L2 Regularization	97.2%	0.968	Better generalization

Figure 13 histogram of Intersection over Union (IoU) scores comparing LIME-highlighted regions with expert-annotated tumor masks. The mean IoU was 0.61 ± 0.12 across 100 test cases, indicating moderate spatial alignment.

## Conclusion, limitations, and future scope

### Summary

The DenseNet169-LIME-TumorNet model in this study does well for research and purposes. It scored above the performance bar at 98.78%, considering the bottom end set by its cousins, namely ResNet50, VGG16, and InceptionV3. By adding LIME, bringing in interpretability, and advancing the naming visualization of decision-making processes, the reliability associated with it becomes critical toward medical diagnostics. All this points to the fact that with the coupling of DenseNet169 and explainable AI techniques, the model has a very strong balance of accuracy as well as interpretation, making it a very good potential instrument for future realistic clinical deployments. This has, however, led to several misclassifications, especially among closely related options. This study presents a unified hybrid architecture combining the transfer learning benefits of DenseNet169 with spatially adaptive parallel CNN branches. This synergy enhances the model’s ability to generalize across tumor shapes and locations, as confirmed by both quantitative and qualitative evaluations.

### Limitations

Despite its strengths, the study has several limitations that should be acknowledged:

- **Dataset Characteristics:** The datasets used may suffer from class imbalance and limited diversity in patient demographics, which could affect the model’s generalizability to broader populations. Additionally, the data may originate from a single institution, limiting external validity.
- **Interpretability Constraints:** While LIME offers local explanations, its reliability may vary across different data distributions and complex, unseen cases, potentially limiting its universal applicability.
- **Scope of Analysis:** The current work focuses solely on classification and does not address tumor segmentation, which is equally important for comprehensive brain tumor diagnosis and treatment planning.
- **Computational Overhead:** Generating LIME explanations during inference introduces additional computational costs, which may impact real-time deployment feasibility in clinical settings.
- **Data Quality Dependence:** The model’s performance and interpretability heavily depend on the quality of MRI scans and the accuracy of annotations, which can vary in clinical practice.

### Future work

Building upon the current findings, several avenues for future research are proposed:

- **Enhanced Generalization:** Implement advanced data augmentation and regularization techniques to improve the model’s robustness and generalization to diverse datasets and patient populations.
- **Hybrid Architectures:** Explore Transformer-based hybrid deep learning models to further enhance feature extraction and classification accuracy, leveraging recent advances in attention mechanisms.
- **Multi-Modal Learning:** Integrate multi-modal data, such as combining MRI images with clinical records and radiological reports, to develop a more comprehensive and robust diagnostic framework.
- **Segmentation Integration:** Extend the model to include tumor segmentation capabilities, enabling more detailed analysis and aiding treatment planning.
- **Real-Time Clinical Deployment:** Focus on optimizing computational efficiency for real-time deployment in hospital environments, facilitating AI-assisted decision-making alongside radiologists.
- **Explainability Improvements:** Investigate alternative or complementary explainability methods to LIME that may provide more stable and global interpretability across varied cases.

These future directions aim to enhance the DenseNet169-LIME-TumorNet model’s clinical applicability, making it a more powerful, interpretable, and practical tool for brain tumor diagnosis.

## Data Availability

Data is provided within the manuscript and cited as Ref^38^.
